# The Mosquito Microbiota: A Key Player in Vector Competence and Disease Dynamics

**DOI:** 10.3390/pathogens13121101

**Published:** 2024-12-13

**Authors:** Vaidas Palinauskas, Salma Kaoutar Abdelali, Alejandro Cabezas-Cruz

**Affiliations:** 1 Nature Research Centre, Akademijos 2, LT-08412 Vilnius, Lithuania; 2 Laboratory of Research on the Improvement and Development of Animal and Plant Production, University of Ferhat Abbas, Setif 19137, Algeria; abdelali.selmakaoutar@gmail.com; 3 ANSES, INRAE, Ecole Nationale Vétérinaire d’Alfort, UMR BIPAR, Laboratoire de Santé Animale, F-94700 Maisons-Alfort, France

Mosquitoes are well-known vectors for a range of pathogens, including *Plasmodium* parasites, which cause malaria in reptiles, birds, and mammals [1,2], as well as arboviruses such as West Nile Virus (WNV) that affect both avian and human populations [3]. Avian malaria has emerged as a significant global concern due to its profound detrimental effects on bird biodiversity and the ecological balance [4,5]. *Plasmodium relictum*, the most widespread avian malaria parasite, infects over 300 bird species and is ranked among the top 100 invasive species globally [6,7]. Its impact is particularly devastating in ecologically sensitive regions such as islands [8]. For example, the introduction of *Plasmodium relictum* and its primary vector, *Culex quinquefasciatus*, to the Hawaiian archipelago caused a severe decline in native bird populations, including the extinction of the Liwi honeycreeper (*Drepanis coccinea*) in certain areas [9]. Understanding the factors that influence disease transmission is crucial, and recent research points to the mosquito microbiota as a pivotal yet underexplored element [10].

The mosquito microbiota [10], a community of bacteria, fungi, and viruses, interacts with the mosquito’s immune system and influences feeding behavior and physiology, impacting pathogen transmission either positively or negatively [11]. This editorial examines the critical role of the mosquito microbiota in the transmission of vector-borne diseases, drawing on insights from Garrigós et al. [12] who focus on WNV. By exploring the complex relationships between mosquito microbiota and pathogens like *Plasmodium* and WNV, we can gain a valuable understanding of how these microbial communities shape vector competence—the ability of mosquitoes to acquire, maintain, and transmit pathogens—potentially unlocking innovative approaches to combating diseases that threaten both biodiversity and public health.

The role of the mosquito microbiota in shaping vector competence has become a focus of growing interest. Studies have revealed the intricate relationships between gut microbial communities and pathogen transmission, shedding light on their influence on disease dynamics [12,13,14,15]. Recently, Garrigós et al. [12] provided a comprehensive review on the role of mosquito microbiota in pathogen transmission, highlighting how bacterial genera such as *Serratia* and *Enterobacter* can enhance the development of WNV in *Culex pipiens*, potentially increasing transmission likelihood. While their review focuses on WNV, it offers valuable insights into potential microbial mechanisms that could similarly affect *Plasmodium* development within mosquitoes. Parallel findings in avian malaria research reveal that the gut microbiota composition correlates with susceptibility and infection intensity in mosquito vectors [7]. In the context of avian malaria, findings indicate that gut microbiota composition correlates with mosquito susceptibility and infection intensity. Martinez-de la Puente [16] showed that microbiota alteration via antibiotics heightened *Plasmodium* prevalence in mosquito saliva, emphasizing the microbiota’s role in pathogen development. Similarly, Aželytė et al. [17] identified specific microbial taxa in *Cx. quinquefasciatus* that directly influence *Plasmodium* transmission success. These studies illustrate the dual nature of the microbiota. While some microbial communities support immune defenses, others facilitate pathogen proliferation, underscoring the complexity of microbiota-mediated vector competence.

Both biotic and abiotic factors shape the composition and function of the mosquito microbiota, which ultimately influence vector competence and the dynamics of disease transmission [18]. Key factors influencing the gut microbiota include breeding site characteristics [19], blood meal types [20], developmental stages [21], and the presence of pathogens [22]. The microbiota is primarily acquired during the larval stage from the surrounding aquatic environment, making larval habitats essential for shaping microbiota diversity. Research by Dickson et al. [20] demonstrated that microbial exposure during larval development can enhance immune priming and alter feeding behavior in adult mosquitoes, thereby impacting their ability to transmit pathogens. Additionally, different mosquito species exhibit considerable microbiota variation. For example, *Culex*, *Aedes*, and *Anopheles* mosquitoes have distinct microbial communities [23,24]. 

Despite these variations, certain microbes are consistently found across species and geographic regions, as noted by Osei-Poku et al. [25], suggesting the presence of a shared microbial core within mosquito populations. Geographical and environmental factors, including local climate, urbanization, and habitat structure, further influence microbiota composition. For instance, Bascuñán et al. [26] found that environmental changes, such as pollution and land-use transformation, indirectly alter vector competence by shaping the microbial communities that mosquitoes encounter. Collectively, these findings highlight the intricate interplay of biological and ecological factors in determining microbiota diversity and stability, underscoring their critical role in modulating mosquito–pathogen interactions.

Given the pivotal role of the mosquito microbiota in vector competence, manipulating the microbiota has emerged as a promising strategy to reduce pathogen transmission. Laboratory studies have demonstrated the feasibility of microbiota interventions within mosquito hosts. For instance, Aželytė et al. [17] showed that microbiota modification through a bird-administered anti-microbiota vaccine could affect *Plasmodium* development within mosquitoes, suggesting the potential for microbiota-based strategies to indirectly disrupt disease transmission. Peixoto et al. [27] emphasized the broader significance of leveraging microbial solutions, advocating for their integration into public health and ecological management strategies. Despite these promising findings, microbiota-targeted approaches remain largely experimental, with several challenges limiting their field application. Effectiveness varies across mosquito species and environmental contexts, necessitating tailored interventions for different ecosystems. Moreover, translating laboratory results to natural populations is complicated by the variability in environmental microbial diversity and mosquito behavior under field conditions. Another critical concern is the ecological impact of microbiota manipulation; while reducing vector competence is desirable, disruptions to natural microbial communities could inadvertently affect mosquito fitness, behavior, or interactions with other species. Future research must address these challenges, focusing on developing safe, species-specific microbiota-based interventions that can be applied effectively in diverse ecological settings.

The mosquito microbiota plays a pivotal role in shaping vector competence and influencing the transmission of diseases such as avian malaria. Factors such as mosquito species, larval habitats, and environmental conditions profoundly affect the composition of these microbial communities, which, in turn, determine pathogen transmission dynamics. In their comprehensive review focusing on WNV, Garrigós et al. [12] highlight the intricate interplay between mosquito microbiota and pathogen development, offering valuable insights into potential control strategies. While their work centers on WNV, the mechanisms they discuss are highly relevant to other mosquito-borne diseases such as avian malaria. This suggests that microbiota-targeted approaches could show great promise for reducing disease transmission across multiple pathogens, though their application demands careful consideration of species-specific variations, ecosystem differences, and potential ecological impacts. Future research should focus on unraveling the complex microbial interactions that influence vector competence under diverse environmental conditions to inform the development of innovative, sustainable interventions. Advancing our understanding of the mosquito microbiota holds the key to mitigating the impacts of avian malaria and safeguarding global biodiversity. 
